# Appendico‐ileal knotting causing small bowel obstruction: A case report

**DOI:** 10.1002/ccr3.5878

**Published:** 2022-05-15

**Authors:** Tewodros Abule, Tsegaye Chebo, Bruke Berhanu Billoro

**Affiliations:** ^1^ Department of Surgery Wachemo University Nigist Elleni Mohamed Memorial Comprehensive Specialized Hospital Hossana Ethiopia; ^2^ Department of Surgery Hawassa University Hawassa Sidama Ethiopia; ^3^ 52988 Department of Clinical Pharmacy Faculty of Pharmacy Near East University Nicosia Northern Cyprus Turkey; ^4^ 52988 Department of Pharmacy College of Medicine and Health Sciences Wachemo University Hosanna Ethiopia

**Keywords:** appendicular knot, band, small bowel obstruction, Wachemo University

## Abstract

Small bowel obstruction (SBO) is a common cause of emergency admission to surgical ward. The etiologies for the majority of these cases include postoperative adhesions and hernia. However, acute appendicitis presenting with SBO due to appendico‐ileal knotting is a very rare and unsuspected condition in an emergency scenarios. We report a case of acute SBO in a 30‐year‐old female patient who was explored, and found to have appendico‐ileal knotting causing SBO.

## Graphical abstract

Small bowel obstruction is one of the most common causes of emergency admission with abdominal pain often requiring surgical intervention. Small bowel obstruction (SBO) is a common cause of emergency admission to surgical ward. The etiologies for the majority of these cases include postoperative adhesions and hernia. However, acute appendicitis presenting with SBO due to appendico‐ileal knotting is a very rare and unsuspected condition in an emergency scenarios.

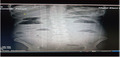

## 1 BACKGROUND

Acute small bowel obstruction is an ever‐increasing clinical problem. Small bowel obstruction is one of the most common causes of emergency admission with abdominal pain often requiring surgical intervention. Successful management depends on comprehensive knowledge of the etiology and pathophysiology of the small bowel obstruction, familiarity with imaging methods, good clinical judgment, and sound technical skills. Intra‐abdominal adhesions related to prior abdominal surgery account for up to 75% of cases of small bowel obstruction followed by hernia.^1^ Appendicular knot, also called as appendicular band syndrome or appendicular tie syndrome, is an extremely rare surgical entity with only a few cases reported so far.^2^, ^3^, ^4^, ^5^, ^6^ It usually presents with intestinal obstruction. The ileum is entrapped by the appendicular knot causing closed‐loop obstruction and may strangulate, leading to small bowel gangrene if not intervened early.^3^, ^4^

Knowledge of this unusual entity is important for clinical suspicion and successful management. Here, we report a case of 30‐year‐old female who presented to us with features of small bowel obstruction and on exploration; there was terminal ileum encirclement due to appendicular knotting. The patient was treated by appendico‐ileal knot releasing and appendectomy.

## 2 CASE REPORTS

A 30‐year‐old female patient presented to surgical emergency department, Wachemo University Nigist Elleni Mohamed Memorial Comprehensive Specialized Hospital on 28/01/2020 G.C with referral paper from Doyogena primary hospital, Southern Ethiopia. The patient presented with crampy periumblical abdominal pain that gradually increased to involve her whole abdomen over 15 days duration. Associated with this she has a history of several episodes of projectile vomiting of ingested matter which latter becomes bilious for 14 days duration.

She has a history of failure to pass both faeces and flatus of 8 days duration. She has also an abdominal distention of 7 days duration and history of loss of appetite of the same duration. Otherwise, there was no history of previous abdominal surgery and known chronic medical illness.

On physical examination, she was acutely sick looking in pain. She was tachycardia to the level of 120 bpm, BP = 117/72, and she was maintaining her saturation on atmospheric oxygen 96% with a respiratory rate of 24 breath per minute. Her temperature was 36.7°C. Her abdomen was grossly distended that moves with respiration with hypoactive bowel sound, hypertympanic percussion note, and mild periumbilical area tenderness on deep palpation. Digital rectal examination reveals normal anal tone with empty rectum. Full blood count shows WBC = 15200/μl with neutrophils (81.1%).

**FIGURE 1:**
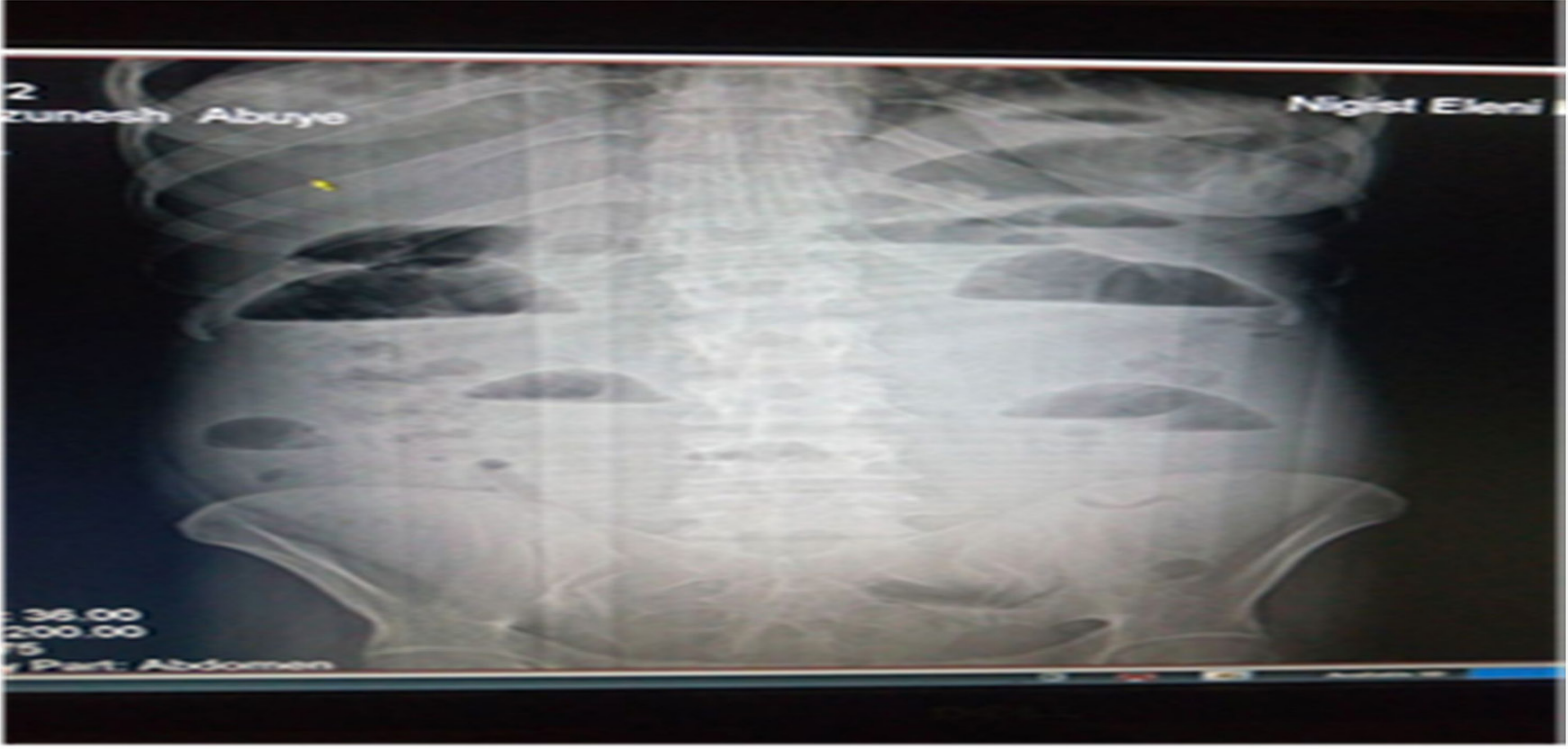
Erect plain abdominal X‐ray showing multiple air fluid levels with paucity of air in the rectum

Plain abdominal X‐ray in erect position showed dilated small bowel loops with multiple air fluid levels and absent rectal gas shadow (Figure 1).

The provisional diagnosis of small bowel obstruction secondary to small bowel volvulus is made and patient is prepared and taken to the operation theater for exploratory laparotomy after detailed informed consent. Under general anesthesia and endotracheal intubation abdomen was entered through midline incision.

**FIGURE 2:**
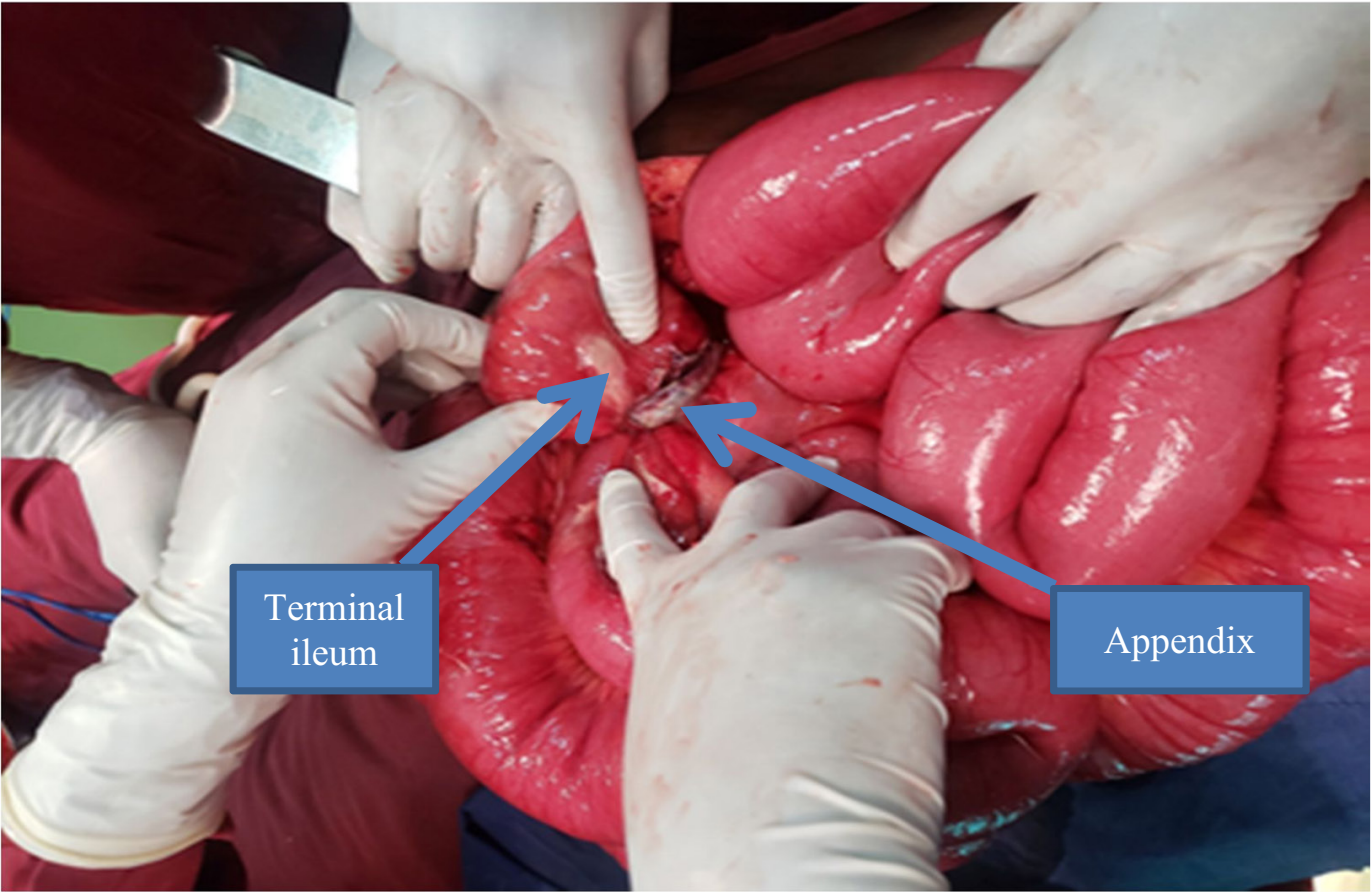
Dilated small bowel loop with appendix causing terminal obstruction

Intra‐operative finding was ~1L of reactive fluid in the general peritoneal cavity and the greater omentum was found migrated to the right lower quadrant. The whole small bowel is dilated and there is a constricting ring around the terminal ileum created by gangrenous appendicitis with its tip adherent to the ileal mesenteric border. There is no sign of ischemia at the site of obstruction and large bowel is collapsed (Figure 2).

**FIGURE 3:**
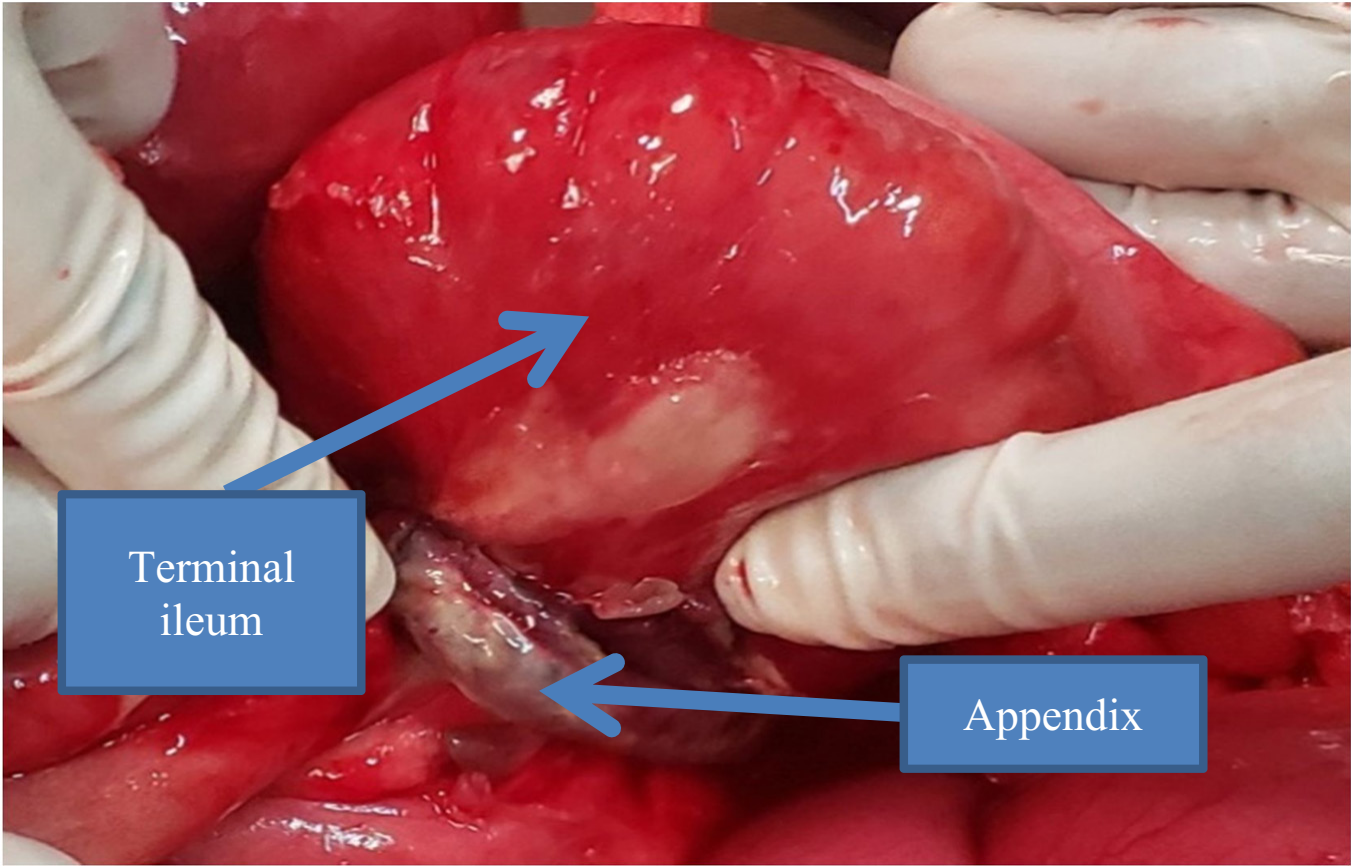
Appendiceal knot formed by adherence of the appendix to part of the mesentery of terminal ileum

The Appendico‐ileal knotting was at a 50cm distance proximal to the ileoceacal valve. The entangled part of the distal ileum was viable with the gangrenous appendix forming knot. The whole length of the appendix was gangrenous involving the base of appendix. The tip of the gangrenous appendix was adherent to the terminal part of the mesenteric border of the ileum forming ring‐like structure with herniation of the terminal 10cm length of the ileum through the ring (Figure 3). The other bowel parts were grossly normal involving the cecum.

The reactive fluid was sucked out. The appendiceal knotting band was released from its attachment to the ileum and appendectomy was done. Retrograde milking of small bowel content was done, peritoneal cavity lavaged with warm saline, and the abdominal wall was closed in layers. The postoperative period was uneventful until the 5th postop day. On the 5th postop day, she developed abdominal distension and loss of appetite but her vital signs were normal. Abdominal examination reveals clean surgical wound with hypertympanic percussion notes and hypoactive bowel sounds. With an impression of postoperative ileus she was managed conservatively by keeping her NPO, IV fluid and NGT decompression. On the next day, the 6th postop day, the abdominal distension subsided and regained her appetite. On the 7th postop day, she was discharged improved with PO analgesics and appointment for follow‐up.

The appendix was not sent for histopathology examination. She was seen subsequently at surgical referral clinic of our hospital and the patient had smooth postoperative course on follow‐up. The surgical wound is clean and surgical stich removed on 14th post‐operative day.

## 3 DISCUSSION

Acute SBO is one of the most common surgical clinical problems. A wide range of etiologies has been reported for small bowel obstruction. Acute appendicitis is a very rare cause of small bowel obstruction. Appendix is a mobile organ and has a variable position. Hence, during appendicitis it has the tendency to get adhered to surrounding structures resulting in mechanical small bowel obstruction and an increased length seems to facilitate the knotting phenomenon.^3^ Appendicular knot or band syndrome is reported in neonates, children, and adults.^2^, ^3^, ^4^, ^5^, ^6^

Appendicitis as a cause of SBO has been known in the literature since 1901 when Lucius Hotchkiss reported three successful surgeries for intestinal obstruction due to appendicitis.^7^ In 1909, Hawks^8^ divided the causes into mechanical and septic appendicitis (adynamic) or a combination of both.

In 2009, Bhandari et al^9^ have reviewed the literature and proposed four categories on the pathology of appendicitis causing obstruction; A dynamic, Mechanical (with or without strangulation), Strangulation and caused by Mesenteric ischemia. Dynamic obstruction or paralytic ileus is undoubtedly the most common type and is due appendicular inflammation spreading to the surrounding structures (caecum, small bowel, or posterior peritoneum). Paralytic ileus caused by appendicular inflammation is the most common cause of intestinal obstruction in acute appendicitis, occurring in 1%–5% cases of appendicitis.^9^

**FIGURE 4:**
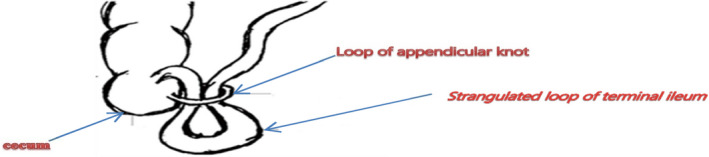
Depiction of appendix wrapping around the loop of ileum. (Reproduced with permission from Menon et al^2^)

Strangulation may result from a long standing closed loop obstruction, which can be due to the long appendix constricting around a loop of the small bowel, or when it is adhered to the surrounding structures and a part of the bowel herniates through the gap (Figure 4).

In 2005, Assenza et al^3^ reported only six cases in the review. Mesenteric ischemia due to appenditis causing intestinal obstruction is the rarest one.

Among the mechanical causes, the vast majority are due to the formation of an appendicular abscess that compresses the loops of the small bowel, and postoperative adhesions that occur years after treatment.^9^ There are two basic situations where the appendix may also cause a mechanical obstruction^10^; an appendicular tip attached to the mesentery surrounding an ileal loop, producing compression of its lumen and the appendicular tip attached to the intestinal serosa, producing the obstruction by direct compression or torsion of the loop.

There are only ten cases reported in the literature reviewed by O’Donnell et al^11^ i.e., a loop obstruction caused by the loop of the appendix attached to the mesentery, in the context of acute appendicitis, which is similar to the one in our case. Soressa et al a study^12^ conducted in Ethiopia revealed intussusception, obstructing/strangulating hernias, small bowel volvulus, post‐op adhesions and small bowel malignancies as the commonest cause of SBO in decreasing frequency. Acute inflammation of the appendix is probably the inciting event of this band formation. The appendix itself may be acutely inflamed, perforated especially at the tip, or it may be completely gangrenous.

This entrapment results not only in intestinal obstruction and strangulation of the entrapped bowel but also in ischemia of the appendix itself due to compression, which was probably the reason of gangrenous appendix in our case.

The reported complications are: intestinal obstruction, volvulus, strangulation of the small bowel, and strangulation of the appendix itself.^2^, ^3^, ^4^, ^5^, ^6^, ^10^ In view of the mobility and variable position of the tip of the appendix, it is possible that the appendix may get adhered to adjacent structures during the phase of inflammation giving rise to the pathology mentioned above. As the formation of an appendiceal knots is a resultant of acute appendicitis, mechanical small bowel obstruction caused by the appendicular knots usually presents a certain period after the episode of acute appendicitis. The paucity of this condition makes it very challenging in making its preoperative diagnosis.

A diagnosis of acute bowel obstruction is made initially based on clinical judgment based on the history and physical examination of the patient. The cardinal symptoms and signs are colicky abdominal pain, vomiting, absolute constipation, and abdominal distension, all of which were present in this patient. Confirmation of bowel obstruction is then usually made with an erect plain abdominal X‐ray, which provides the surgeon with several information including whether there is small or large bowel obstruction and the degree of obstruction. In the present case, dilated loops of the small bowel indicated acute small bowel obstruction. The straight X‐ray of the abdomen may be helpful in the diagnosis of small bowel obstruction, but it often fails to identify the etiology. In the early inflammation phase, CT (Computerized Tomography) may help to clinch the diagnosis. After resolution of appenditis, its role is very limited.^2^, ^3^, ^9^

Thorough history and clinical examination, imaging findings, and high index of suspicion may help in diagnosis. Diagnostic laparoscopy may be a valuable option. Treatment is straightforward and depends on intraoperative findings.

The management depends on the extent of the strangulation and the part of the bowel involved. A midline laparotomy is advisable to rule out other causes of obstruction. It may range from appendectomy to right hemicolectomy.

It may require small bowel or ileocaecal resection when there is strangulation. Release of the appendiceal band and appendectomy is adequate when the herniated bowel is viable and if intervened early, as it was in our case. Resection of the nonviable intestine and anastomosis or stoma creation is necessary in the presence of nonviable gut. Closed loops and strangulating obstruction of the small bowel are serious lesions that require emergency surgery. An accurate and early diagnosis of intestinal strangulation is essential in patients with small bowel obstruction to minimize the risk of morbidity and mortality. Delayed operation potentially results in high mortality.

## 4 CONCLUSION

Small bowel obstruction is a rare complication of acute appendicitis. Appendiceal knot is a rare cause of this and very few cases have been reported. As a surgeon dealing with acute abdomen in day‐to‐day practice, knowledge about the diagnosis and management of this rare scenario is very helpful. A high index of clinical suspicion is of utmost importance in identifying and correctly managing this rare condition.

## AUTHOR CONTRIBUTIONS

AT and TC: performed the surgery, Writing—Original Draft. BB: Writing—Review and editing it critically for intellectual content. All authors read and approved the final manuscript.

## CONFLICTS OF INTEREST

The authors declare that they have no conflicts of interest.

## ETHICAL APPROVAL

The report conformed to the principles outlined in the Declaration of Helsinki and was approved by the Hospital Medical ethics and research ethics committee at Wachemo University Nigist Elleni Mohamed Memorial Comprehensive Specialized Hospital, Ethiopia.

## CONSENT

Written consent has been obtained from the patient for publication of this report.

## Data Availability

Data sharing not applicable to this article as no datasets were generated or analysed during the current study.
